# Brain organoids: a new paradigm for studying human neuropsychiatric disorders

**DOI:** 10.3389/fnins.2025.1699814

**Published:** 2025-11-03

**Authors:** Yi Sun, Wei Pan

**Affiliations:** ^1^Jiangsu Key Laboratory of Immunity and Metabolism, Department of Pathogenic Biology and Immunology, Xuzhou Medical University, Xuzhou, Jiangsu, China; ^2^The First Clinical Medical College, Xuzhou Medical University, Xuzhou, Jiangsu, China; ^3^National Experimental Teaching Demonstration Center of Basic Medicine, Xuzhou Medical University, Xuzhou, Jiangsu, China

**Keywords:** brain organoids, organoid assembly, brain development, neurodevelopmental disorders, neurodegenerative disorders, psychiatric disorders

## Abstract

Understanding human brain development and dysfunction is a major goal in neurobiology. Compared with traditional 2D models and animal models, brain organoids technology based on induced pluripotent stem cell (iPSC) constructs can more accurately recapitulate the developmental process of the human brain and simulate the characteristic phenotypes of neurological diseases in recent years. This technology is expected to change our understanding of human brain development, while providing a fresh perspective on elucidating the pathogenesis of inherited and acquired brain diseases. This article reviews the development and recent advances in brain organoids, explores their use in neuropsychiatric disorders, from neurodevelopmental to neurodegenerative and psychiatric diseases, while also outlining the challenges facing the technology. We conclude that these advances not only enhance our understanding of human-specific brain development and disease mechanisms, but also accelerate the translation of brain organoid technology into personalized medicine and drug discovery.

## 1 Introduction

The human brain is the most structurally and functionally sophisticated organ in biological systems, composed of highly diverse cell populations. Due to technical limitations and ethical considerations, researchers face significant challenges in obtaining human brain tissue samples with full physiological activity, which severely hinder the exploration of functional mechanisms within the human nervous system. Currently, neuroscience research primarily employs two experimental systems: two-dimensional (2D) cell models and animal models. Several commonly used human neuronal cell lines, such as SH-SY5Y and IMR-32 (neuroblastoma) (Shipley et al., 2016; Zitta et al., 2016), and LUHMES cells (immortalized neuronal cell line) (Renner et al., 2024), have been utilized as *in vitro* neuronal models for analyzing cellular phenotypes. While these cell lines can proliferate indefinitely under *in vitro* culture conditions, they lack sufficient functional and neural maturity to mimic the human brain’s three-dimensional spatial architecture, complex intercellular communication networks, and dynamic microenvironmental regulatory mechanisms. Simultaneously, interspecies biological differences, inadequate simulation of pathological phenotypes, and biases in microenvironmental and immune interactions significantly constrain the application of animal models. Furthermore, other model systems, such as neural stem cells (NSCs) and primary brain cell cultures, have been widely utilized. NSCs possess the capacity to differentiate into both neuronal and glial lineages, offering greater differentiation potential than immortalized cell lines. However, they may suffer from epigenetic drift upon long-term expansion *in vitro* and struggle to recapitulate the complex region-specific epigenetic landscapes of the developing brain (Ziffra et al., 2021; Van den Ameele et al., 2020). On the other hand, primary cultured brain cells, especially from human sources, have limited sources and exhibit significant donor-to-donor variability. Their short culture lifespan and difficulties in genetic manipulation collectively hinder their utility for studying human-specific developmental dynamics (Trevino et al., 2020) and conducting large-scale drug screening.

Brain organoids effectively address the aforementioned limitations. Brain organoids are three-dimensional, self-organizing and miniaturized *in vitro* culture models. By recapitulating certain key aspects of human brain development, they can generate a diversity of cell types, including neurons and glia relevant to specific brain regions. This 3D architecture mimics the complex cellular composition, spatial organization, and cell–cell interactions found in the developing brain to a degree that is unattainable in traditional 2D cell cultures (Lancaster and Knoblich, 2014). They more accurately reflect human tissue, offering new possibilities for investigating brain development, modeling neurological disorders, and conducting drug screening (Xie et al., 2025). This paper will outline various research methods and examples of brain organoids to discuss their latest developments and applications in studying diverse neurological conditions, including neurodevelopmental disorders, neurodegenerative diseases, and psychiatric disorders.

Induced pluripotent stem cells (iPSCs) are generated by reprogramming somatic cells (e.g., skin fibroblasts or blood cells) back into an embryonic-like state. This revolutionary technology, typically achieved by introducing a set of defined transcription factors (such as OCT4, SOX2, KLF4, and c-MYC), endows these cells with the capacity for unlimited self-renewal and the potential to differentiate into virtually any cell type of the body (Takahashi and Yamanaka, 2023). Crucially, as iPSCs can be derived directly from patients, they provide an unparalleled platform for modeling human diseases and advancing personalized medicine (Rowe and Daley, 2019). In 2013, Jürgen Knoblich and Madeline Lancaster employed Matrigel to simulate the microenvironment of developing brain tissue. By utilizing a rotating cell culture system to promote uniform distribution of metabolic substances and gas exchange, while supplementing specific cytokines regulating neural development, they achieved the first successful differentiation of iPSCs into functional brain organoids (Lancaster et al., 2013). This model contained interconnected functional units resembling the forebrain, choroid plexus, hippocampus, and prefrontal cortex, exhibiting cellular composition and tissue architecture similar to that of the developing fetal brain (Lancaster and Knoblich, 2014). Subsequently, by combining different small molecules and growth factors, researchers successfully constructed multiple brain organoid models, including the cerebral cortex (Cao et al., 2023), basal ganglia (Hunt et al., 2023), hypothalamus (Kasai et al., 2020), midbrain (Nishimura et al., 2023), cerebellum (Nayler et al., 2021), spinal cord (Guan et al., 2025), and striatum (Miura et al., 2020), reproducing the developmental processes of specific brain regions.

In recent years, diverse protocols have been established to enhance the reproducibility and regional specificity of brain organoids. These protocols vary in the starting cell types, the use of patterning factors, and morphogenetic guidance strategies, aiming to model either whole-brain development or specific brain regions. For instance, the pioneering protocol from the Knoblich lab generates whole-brain organoids containing multiple brain region identities (Lancaster and Knoblich, 2014), whereas protocols from the Pasca lab utilize exogenous morphogens to precisely generate region-specific organoids with dorsal or ventral forebrain characteristics (Sloan et al., 2018; Table 1). Hendriks et al. (2024) developed brain organoids (FeBOs) directly from human fetal brain tissue, offering novel approaches for investigating the development and therapeutic interventions for brain-related diseases, including brain cancer (Table 1). Ramani et al. (2024) developed the “Hi-Q brain organoid” culture method, bypassing the traditional embryoid body (EB) stage. This approach directly induces iPSCs to differentiate into neurospheres, precisely controlling their size using custom uncoated microplates. This eliminates the size inconsistencies and differentiation abnormalities associated with the EB stage. This method enables the generation of hundreds of high-quality brain organoids per batch with minimal activation of cellular stress pathways, supporting cryopreservation and recultivation (Table 1). The choice of protocol depends on the research objective—whether to explore global brain organization or to accurately model disorders associated with specific brain circuits. Furthermore, several studies have achieved functional integration of human brain organoids within the brains of living rodents, thereby extending organoid culture lifespan and generating more mature functional neurons (Nayler et al., 2021; Dong et al., 2021; Revah et al., 2022).

**Table 1:** Comparison of representative brain organoid generation protocols.

Protocol/Lab	Key features	Advantages	Disadvantages/Limitations	References
Whole-Brain/Unpatterned Organoids (Knoblich/Lancaster)	• Relies on cellular self-organization • Embedded in Matrigel • Uses rotating bioreactors	• Models interactions between multiple brain regions • No exogenous patterning factors required • Suitable for studying global developmental events	• High batch-to-batch variability • Uncontrolled regional composition • Frequent necrotic core formation	Lancaster and Knoblich (2014)
Region-Specific/Patterned Organoids (Pasca et al.)	• Uses small molecule morphogens • Directed differentiation into specific brain regions • Precise control of developmental pathways	• High regional consistency and reproducibility • Good cellular purity • Ideal for studying region-specific disorders	• Sacrifices whole-brain complexity • Requires pre-definition of target brain region • Demands precise timing and concentration of morphogens	Sloan et al. (2018)
Fetal Brain Organoids (FeBOs) (Hendriks et al.)	• Direct use of fetal brain tissue • Preserves native microenvironment • Long-term self-expansion	• Maintains cellular diversity of primary tissue • Preserves *in vivo* spatial characteristics • Unique model for development and brain cancer	• Extremely limited tissue availability • Significant ethical considerations • Cannot study early neurodevelopmental events	Hendriks et al. (2024)
Assembloids (Pasca et al.)	• Assembly of organoids from different regions • Models inter-regional connectivity • Studies cell migration and projections	• Enables study of long-range neuronal connections • Reveals mechanisms of brain region interactions • Models complex neural circuits	• Higher technical complexity • Assembly efficiency requires optimization • Fusion consistency needs improvement	Andersen et al. (2020)
Micropatterned/Bioengineered Organoids (Song et al.)	• Uses micropatterned substrates • Precise control of initial size and shape • Engineered control of morphogenesis	• Excellent initial uniformity • Effectively reduces necrotic cores • Suitable for quantitative studies	• Requires specialized equipment • Demands bioengineering expertise • Limited adoption in standard biology labs	Gillett et al. (2022)
Hi-Q Brain Organoids (Ramani et al.)	• Bypasses embryoid body stage • Uses custom uncoated microplates • Precise control of neurosphere size	• High reproducibility and consistency • Minimal activation of cellular stress pathways • Supports cryopreservation and large-scale screening	• Relatively new protocol • Long-term developmental potential requires further validation • Broad applicability needs independent verification	Ramani et al. (2024)

Region-specific brain organoids can generate highly uniform populations of progenitor cells and neurons by regulating developmental signaling pathways, significantly reducing heterogeneity among organoids. However, this approach cannot study interactions between different brain regions. To overcome this limitation, scientists developed the “assembloid (a complex multi-region organoid assembly)” technique. By assembling organoids from different brain regions, this approach further simulates more complex neurodevelopmental processes and reveals subtle pathological changes in neurological disorders. Based on this technology, cortical-striatal assembloids (Reumann et al., 2023), cortical-thalamic assembloids (Patton et al., 2024), and midline assembloids (Onesto et al., 2024) have been established to simulate long-range axonal connections, offering innovative platforms for investigating the development and function of complex neural circuits. With deepening research into the nervous system, dynamic interactions between neuronal and non-neuronal cells have been shown to play equally important roles in neurodevelopment and disease. Microglia, the brain’s resident macrophages, play an essential role in regulating neural circuits, maintaining homeostasis, and monitoring immune function. Their dysfunction is mechanistically linked to neurodegenerative diseases and psychiatric disorders, including schizophrenia (Sun et al., 2025; Lukens and Eyo, 2022). Several studies have attempted co-culture of *in vitro* differentiated microglia with brain organoids (Zhang and Cui, 2021) to investigate their roles in physiological and pathological processes. However, traditional brain organoid models exhibit significant vascularization defects, leading to necrotic cores, abnormal oxygen/nutrient gradients, and metabolic waste accumulation, which limit their growth, maturation, and immune function. Sun et al. (2022) constructed brain organoids with functional vascular networks by fusing induced vascular organoids and brain organoids. This assembly successfully mimicked the functional blood–brain barrier (BBB) structure and the phagocytic function of microglia. The advent of microfluidic technology has also brought new breakthroughs to brain organoid research. It enables precise control of the cellular microenvironment, promotes the formation of vascular networks, and allows for real-time dynamic monitoring of neurons (Wang et al., 2019). However, current vascularized brain organoid models cannot fully replicate *in vivo* conditions and require further optimization of induction conditions to enhance developmental stability. Other examples of co-assembly include co-culturing patient-specific glioblastoma organoids with cortical organoids to simulate glioblastoma (Bhaduri et al., 2020), and integrating brain organoids with intestinal organoids to model neuropathic disorders related to the brain-gut axis (Wang et al., 2020). Thus, the co-assembly approach expands the application boundaries of brain organoids in both fundamental research and clinical translation, while also offering potential models for drug discovery and therapeutic interventions.

To improve brain organoid culture techniques and mitigate variability introduced by manual manipulation, researchers are optimizing culture systems and environments by integrating multiple engineering technologies, including bioprinting and bioreactors. The synergistic application of these technologies holds promise for significantly enhancing the functional maturity and experimental reproducibility of brain organoids. Emerging analytical techniques, including whole-cell patch-clamp recording, calcium imaging, electrochemical analysis, and optogenetics, are also employed to systematically document morphological features and developmental dynamics of brain organoids. This provides crucial theoretical foundations for developing treatments targeting related neurological disorders.

Given their unique advantages in simulating the three-dimensional structure and developmental processes of the human brain, brain organoids have emerged as a powerful model for studying neurological disorders. The following section will systematically elaborate on the specific applications of brain organoids in the study of three major categories of neurological disorders.

## 2 Neurodevelopmental disorders

**Figure 1:**
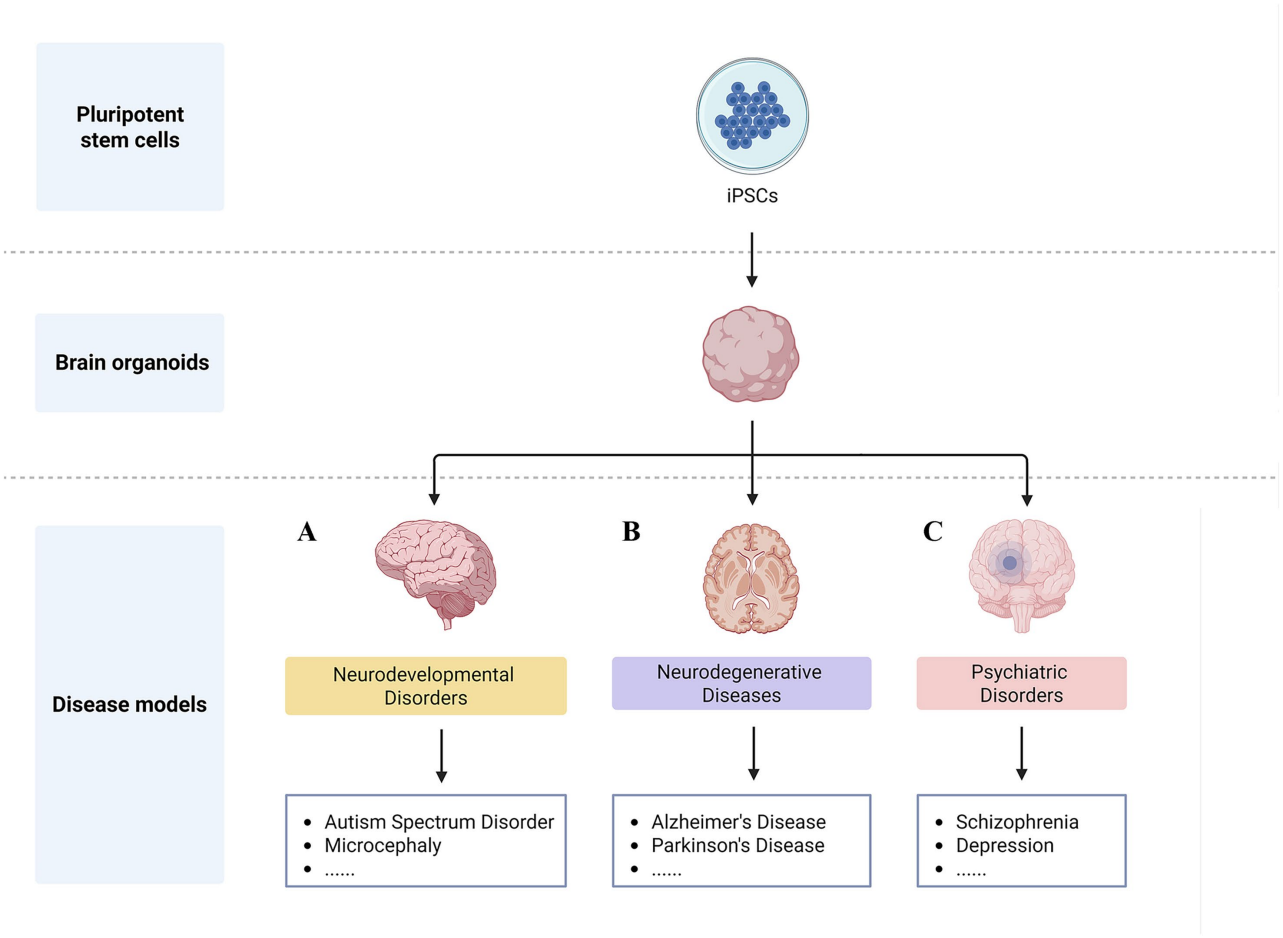
Schematic overview of brain organoid applications in neuropsychiatric disorders. A. Neurodevelopmental disorders. Brain organoids serve as a valuable model system for investigating neurodevelopmental disorders, including autism spectrum disorder and microcephaly. B. Neurodegenerative disorders. Brain organoids make it possible to study neurodegenerative diseases such as Alzheimer’s disease (AD) and Parkinson’s disease (PD). C. Psychiatric disorders. Brain organoids provide a new direction for research on schizophrenia, depression and other mental disorders.

Neurodevelopmental disorders (NDDs) are a class of conditions affecting brain development and function caused by disruptions in the neurodevelopmental process, exhibiting broad genetic and phenotypic variability (Figure 1). NDDs include autism spectrum disorder (ASD), attention deficit hyperactivity disorder (ADHD), and intellectual disability. NDDs exhibit diverse clinical phenotypes, with a particularly pronounced phenomenon of comorbidity—for example, cortical developmental abnormalities are commonly observed in both ASD and intellectual disability patients. Elucidating shared pathogenic mechanisms across different NDDs holds critical value for understanding disease comorbidity and establishing effective treatment strategies. Brain organoids and their derived assembloid systems, being of human genomic origin, enable the faithful reproduction of disease phenotypes triggered by intrinsic (genetic) or extrinsic (environmental) factors. This provides a superior model for investigating the pathogenic mechanisms of NDDs and potentially developing novel therapeutic interventions.

### 2.1 Autism Spectrum disorder

ASD is a neurodevelopmental disorder characterized by core symptoms encompassing social communication deficits and repetitive behaviors, influenced by multiple genetic and environmental factors (Table 2). ASD stands as the most prevalent neurodevelopmental disorder, characterized by remarkable genetic and phenotypic heterogeneity. Indeed, over 1,000 genes have been implicated in its etiology, contributing to its diverse clinical manifestations (Xiong et al., 2019). Well-studied monogenic syndromes include fragile X syndrome (FXS) caused by *FMR1* mutations and Rett syndrome resulting from *MECP2* mutations. Autopsy studies and neuroimaging data reveal multiple pathological alterations in ASD patients, including impaired synaptic plasticity and neural circuit assembly (Hodis et al., 2025), structural disorganization in specific brain regions, neuronal morphological abnormalities, and dysregulated glial cell proportions (Courchesne et al., 2024). To investigate the cortical developmental abnormalities observed in ASD patients, Courchesne *et al*. constructed brain cortical organoids from iPSCs derived from ASD patients. They found that patient organoids exhibited significantly increased volume compared to controls, and this increase correlated positively with the severity of social impairments (Li et al., 2023). Further mechanistic studies revealed that this abnormal growth was associated with overexpression of the transcription factor FOXG1. By manipulating FOXG1 expression in the organoid model, the researchers confirmed that its upregulation directly led to abnormal proliferation of GABAergic interneurons. This provides a key molecular mechanism for understanding the developmental origins of excitatory/inhibitory imbalance in cortical circuits of ASD patients (Manoli and State, 2021). Han et al. (2025) experimentally observed that ASD is associated with premature differentiation of neural stem cells due to abnormal activation of the FABP7/MEK pathway. These findings jointly indicate that ASD correlates with alterations in multiple genes and pathways, suggesting that targeting these pathways may represent a promising therapeutic approach for the disorder. To directly address the genetic complexity of ASD, the CHOOSE (CRISPR-based High-throughput Organoid Screening and sequencing) system developed by Li Chong’s team in 2023 innovatively achieved parallel editing and functional assessment at single-cell resolution for 36 ASD high-risk genes within brain organoid models (Li et al., 2023). This technology established the first ASD genotype–phenotype atlas spanning neurodevelopment, thereby identifying the cellular fates or developmental processes most susceptible to different ASD risk genes. This offers an unprecedented opportunity to uncover the core downstream pathways driving the ASD phenotype.

**Table 2:** Summary of key technical parameters in brain organoid-based disease models.

Disease category	Specific disease	Key research gene/factor	Organoid type/brain region	Cell source	Core pathological phenotype	References
Neurodevelopmental Disorders	Autism Spectrum Disorder	FOXG1, FABP7	Forebrain/Cortical Organoids	ASD Patient iPSC	Increased organoid size, altered NPC/neuron fate determination, excitation/inhibition imbalance	Onesto et al. (2024), Sun et al. (2025), and Lukens and Eyo (2022)
	Microcephaly	CDK5RAP2, WDR62	Whole Brain Organoids	Gene-Edited iPSC	Premature NPC differentiation, spindle orientation defects, decreased organoid size	Sun et al. (2022) and Manoli and State (2021)
		IER3IP1	Whole Brain Organoids	Gene-Edited iPSC	ER stress, unfolded protein response activation, apoptosis	Hodis et al. (2025)
		Zika Virus	Whole Brain Organoids	hESC/iPSC	Disrupted neuroepithelial structures, extensive NPC apoptosis	An et al. (2022)
Neurodegenerative Diseases	Alzheimer’s Disease	APP/PSEN1 (APPNL-G-F)	Whole Brain/Cortical Organoids	FAD Patient iPSC/Gene-Edited ESC	Aβ plaques, hyperphosphorylated tau, neuronal death	Eichmüller et al. (2022) and Arber et al. (2021)
		APOE4	Cortical Organoids/Cortical-Hippocampal Assembloids	Isogenic iPSC	Reduced Aβ clearance, exacerbated tau propagation, aberrant microglial response	Jin et al. (2022), Hou et al. (2019), Chi et al. (2018), and Nassor et al. (2020)
	Parkinson’s Disease	LRRK2 (G2019S)	Midbrain Organoids	Patient iPSC/Gene-Edited iPSC	Dopaminergic neuron degeneration, α-synuclein aggregation, impaired mitophagy	Blanchard et al. (2022)
		DNAJC6	Midbrain Organoids	Gene-Edited iPSC	Dopaminergic neuron degeneration, *α*-synuclein aggregation, lysosomal dysfunction	Zhao et al. (2020)
Neuropsychiatric Disorders	Schizophrenia	22q11.2 Microdeletion	Thalamic Organoids/Cortical-Thalamic Assembloids	Patient iPSC/Gene-Edited iPSC	Thalamic neuron axon overgrowth (FOXG2-dependent), aberrant circuit connectivity	Morrone Parfitt et al. (2024)
		PCCB	Forebrain Organoids	Gene-Edited iPSC	Impaired TCA cycle, reduced GABA levels, energy metabolism deficits	Pena et al. (2024)
	Depression	HTR2C	Ventral Forebrain Organoids/GABAergic Neurons	Patient iPSC	Reduced dendritic complexity, aberrant neuronal electrical activity, weakened calcium activity	Deng et al. (2024)
		WFS1	Hypothalamic Organoids/Whole Brain Organoids	Gene-Edited Animal Model/iPSC	Metabolic dysregulation, depression-like behavior, altered neuroplasticity	Trubetskoy et al. (2022)
		circFKBP8 (5S,6)	Cortical Organoids	Gene-Edited iPSC	Dysregulated glucocorticoid receptor nucleocytoplasmic transport, HPA axis abnormalities	Stankovic et al. (2024)

### 2.2 Microcephaly

Microcephaly is a common pediatric neurodevelopmental disorder characterized by significantly reduced brain volume compared to age-matched normal standards. It is frequently accompanied by multiple organ dysfunction, including epilepsy, intellectual disability, visual and auditory impairments, and congenital heart disease (Table 2). The pathogenesis of microcephaly involves multiple key genes, including those currently identified: *CDK5RAP2* (Erdogan et al., 2025), *CPAP* (An et al., 2022), *KATNB1* (Peluso et al., 2021), *aspartyl-tRNA synthetase 1 (NARS1)* (Manole et al., 2020), *ASPM* (Almeida et al., 2025), *IER3IP1* (Esk et al., 2020), *KNL1* (Fellows et al., 2024), *PTEN* (Dhaliwal et al., 2021), and *WDR62* (Verloes et al., 2022). In brain organoid models, knockout of *CDK5RAP2* and *WDR62* genes leads to premature differentiation of neural progenitor cells (NPCs), misdirected spindle orientation, disrupted symmetric division balance, and ultimately significant organoid volume reduction (Erdogan et al., 2025; Verloes et al., 2022). In *IER3IP1*-knockout brain organoids, endoplasmic reticulum (ER) stress and the unfolded protein response (UPR) pathway are hyperactivated, causing ER morphological and functional disruption. Concurrently, genes involved in the ER-associated protein degradation (ERAD) pathway show specific upregulation, further indicating disrupted protein homeostasis within the ER (Esk et al., 2020). These studies reveal that maintaining the stability of the neural progenitor cell pool is fundamental to normal brain development, and preserving protein homeostasis is crucial for the survival of neural progenitor cells and brain growth. Furthermore, mutations or deletions in human genes associated with positive regulation domain protein 16 (PRDP16), ubiquitin-like modification activator 5 (UMA5), and proto-cadherin (PCDH) have also been implicated in microcephaly (Suresh et al., 2023; Chen et al., 2024; Rakotomamonjy et al., 2023). In addition to simulating genetic factors, brain organoid models can also effectively mimic the pathological mechanisms of conditions such as microcephaly caused by Zika virus infection. The virus inhibits brain growth by directly damaging neural epithelial structures, inducing massive apoptosis of neural progenitor cells, and disrupting the expression of cell cycle regulatory genes (Metzler and Tang, 2024). In summary, human brain organoid models enable researchers to deeply analyze the mechanisms of key pathogenic factors such as genetic mutations and pathogenic microbial infections, while providing a common target for intervention: protecting and maintaining the health and normal proliferation of neural progenitor cells.

Additionally, brain organoid models have been successfully applied to other neurodevelopmental disorders, such as macrocephaly (Dang et al., 2021), Down’s Syndrome (DS) (Jin et al., 2022), Angelman Syndrome (AS) (Sen et al., 2020), neuronal migration defects (Meng et al., 2023), tuberous sclerosis (Eichmüller et al., 2022), and Timothy syndrome (Birey et al., 2022). These investigations have identified abnormalities in key molecular pathways, thereby identifying multiple potential molecular therapeutic targets and multidimensional repair strategy systems, laying a crucial foundation for clinical translational research.

## 3 Neurodegenerative diseases

The clinical subtypes of neurodegenerative diseases (NDD) are primarily categorized as follows: Alzheimer’s Disease (AD), Huntington’s Disease (HD), Parkinson’s Disease (PD), amyotrophic lateral sclerosis, and multiple sclerosis (Figure 1). Research indicates that aging, genetic factors, abnormal protein aggregation, and dysregulated programmed cell death are the primary causes of NDD (Hou et al., 2019; Chi et al., 2018). The establishment of organoids modeling distinct brain regions, including the whole brain (Nassor et al., 2020), forebrain (Arber et al., 2021), midbrain (Toh et al., 2023), striatum (Reumann et al., 2023), and sensorimotor cortex (Liu et al., 2022)—provides highly biomimetic experimental platforms for exploring NDD pathological mechanisms. These platforms enable researchers to dynamically monitor disease-related gene expression, observe cellular interaction networks, and track conformational changes in pathological proteins.

### 3.1 Alzheimer’s disease

As the neurodegenerative disorder with the highest incidence rate (Livingston et al., 2020), AD is characterized by the extracellular oligomerization of *β*-amyloid (Aβ) into amyloid plaques and the intracellular hyperphosphorylation and aggregation of tau protein into neurofibrillary tangles (Hanseeuw et al., 2019; Table 2). Using brain organoid models derived from iPSCs, researchers have successfully reproduced AD-associated features, including amyloid plaque formation, abnormal tau phosphorylation, apoptosis, synaptic loss, and stress granule formation (Zhao et al., 2020). Compared to common alleles like *APOE2* and *APOE3*, apolipoprotein E4 (*APOE4*) represents the strongest genetic risk factor for sporadic AD. A recent study indicates it may accelerate neurodegeneration by disrupting myelin formation through interference with oligodendrocyte cholesterol metabolism (Blanchard et al., 2022). Furthermore, compared to *APOE3*, *APOE4* exhibits reduced capacity for Aβ plaque clearance (Liu et al., 2022). Brain organoid assemblies, by integrating organoids from different brain regions (e.g., the cortex-hippocampus system), can simulate tau protein propagation in AD, the transregional propagation of tau from the hippocampus to the neocortex, and the regulatory role of *APOE4* in microglia (Knopman et al., 2021; van der Kant et al., 2020; Koutsodendris et al., 2023). A recent study employed *CRISPR-Cas9* gene editing to integrate the familial amyloid precursor protein mutant (APPNL-G-F) into human embryonic stem cells. Through directed differentiation, APPNL-G-F brain organoids reproducing AD pathology were generated, revealing the rare homozygous *APOE3* mutation (*APOE3ch*) as a potential protective factor against AD (Liu et al., 2024). In 2014, Choi *et al*. pioneered the utilization of AD brain organoids as a drug screening platform, demonstrating that β−/*γ*-secretase modulators simultaneously reduce Aβ deposition and tau pathology (Choi et al., 2014), providing crucial experimental evidence for AD therapeutics. Interestingly, proteomic analysis of AD-mimetic brain organoids revealed that hallucinogens can attenuate Aβ plaque deposition and inhibit tau hyperphosphorylation by activating 5-HT2A receptors (Androni et al., 2025), making hallucinogens as promising drug candidates for treating AD.

### 3.2 Parkinson’s disease

PD is the most prevalent movement disorder, characterized by aberrant aggregation of *α*-synuclein (α-syn) into Lewy bodies and selective loss of dopaminergic neurons in the substantia nigra (SN) (Yemula et al., 2022; Lal et al., 2024; Table 2). Mutations in genes, such as SNCA, PARK2, PINK1, and LRRK2, are closely associated with PD risk (Singleton et al., 2013). Wulansari et al. (2021) introduced mutated *DNAJC6* genes into human midbrain organoids, successfully establishing an *in vitro* model of PD. This model reproduces multiple key pathological features, including degenerative changes in SN dopaminergic neurons, abnormal α-syn aggregation, enhanced neuronal electrical activity, and mitochondrial and lysosomal dysfunction. Midbrain organoids provide an effective platform for investigating the development and pathology of the dopaminergic system, particularly in PD. Recently, in LRRK2-G2019S mutant midbrain organoids, α-synuclein aggregation was observed to trigger abnormal mitochondrial autophagy accompanied by neurodevelopmental defects (Fiorenzano et al., 2025). These findings suggest that mitochondrial and lysosomal dysfunction may form a vicious cycle, impairing protein degradation capacity, while the compromised clearance system further leads to the accumulation of abnormal proteins. In addition, a study on the DJ1-related PD model further demonstrates that abnormal protein glycosylation and widespread protein aggregation constitute the fundamental pathology of familial PD (Morrone Parfitt et al., 2024). Crucially, this breakdown of protein homeostasis is directly linked to impaired lysosomal proteolytic function in astrocytes, indicating that glial cells play a vital role in maintaining protein homeostasis.

Moreover, brain organoids offer valuable insights into exploring the pathogenesis and potential intervention strategies for neurodegenerative diseases, including ALS (Wang et al., 2019) and HD (Szebényi et al., 2021). Currently, brain organoids can effectively reproduce key pathological features of multiple NDDs, making them an ideal platform for screening potential therapeutic drugs. Taking PD as an example, studies have confirmed that LRRK2 inhibitors can improve dopaminergic neuron abnormalities in midbrain organoids harboring the LRRK2-G2019S mutation, demonstrating certain therapeutic potential (Pena et al., 2024). However, the loss of blood–brain barrier integrity in advanced NDD stages (Liu et al., 2024), the highly complex specific pathological mechanisms of different neuronal subpopulations, and the dynamic interactions between cells within neural networks collectively constrain the application of brain organoids in NDD research.

## 4 Psychiatric disorders

Psychiatric disorders exhibit significant clinical differences from neurological diseases. These conditions typically lack clear neurological lesions, their diagnosis does not rely on brain imaging, electroencephalography, or cerebrospinal fluid testing, and they lack a unified pathophysiological mechanism (Figure 1). They primarily include schizophrenia (SCZ), depression, and bipolar disorder. Recently, Deng et al. (2024) developed a large-scale parallel reporter gene analysis system (lentiMPRA) based on lentiviral vectors and conducted systematic functional assessments of non-coding regulatory elements and their genetic variants in brain organoid models. Experimental results revealed that certain genetic variants associated with mental disorders exhibit differential enhancer activity between alleles, providing novel perspectives for elucidating the pathogenesis of mental disorders and neurodevelopment.

### 4.1 Schizophrenia

SCZ is a mental disorder with polygenic inheritance characteristics, whose etiology involves a complex interplay of genetic susceptibility and environmental risk factors (Table 2). Genetic studies, such as genome-wide association studies (GWAS), have identified over 100 common single nucleotide polymorphisms (SNPs) loci significantly associated with SCZ (Trubetskoy et al., 2022). Epidemiological studies indicate that prenatal stress (Stankovic et al., 2024), fetal vitamin D deficiency (Jaholkowski et al., 2023), and abnormal maternal immune activation (Han et al., 2021) constitute risk factors for SCZ onset. A recent study has revealed that these risk factors may share a common pathophysiological pathway: developmental abnormalities in cerebral vascular endothelial cell function and blood–brain barrier integrity (Stankovic et al., 2024). Shin et al. (2024) discovered in thalamic organoids that 22q11.2 microdeletions upregulate *FOX2* gene expression in thalamic neurons, leading to axonal overgrowth. *FOX2*-dependent axonal abnormalities correlate with developmental deficits in SCZ, suggesting potential therapeutic targets. Recently, researchers constructed brain organoids from patient-derived pluripotent stem cells, directly demonstrating functional neural network deficits in SCZ. These deficits manifest as synaptic formation and transmission abnormalities, leading to increased GABAergic drive (Heider et al., 2024). It is interesting that another study found that knocking out the *β*-subunit of the acyl-CoA carboxylase (PCCB) gene in human forebrain organoids inhibits the tricarboxylic acid cycle, leading to reduced GABA levels and increased SCZ risk (Zhang et al., 2023). These findings suggest that GABAergic pathways and mitochondrial function are implicated in the pathogenesis of SCZ.

### 4.2 Depression

Depression, a globally prevalent mental disorder, typically manifests as persistent anhedonia, disrupted sleep rhythms, and impaired cognitive function (Li et al., 2021; Table 2). Over the past five decades, research on depression has primarily focused on monoamine neurotransmitter systems such as serotonin (5-HT), dopamine (DA), and norepinephrine (NE). However, the pathogenesis of depression is more complex, involving multiple biological abnormalities. These include neurotransmitter dysfunction, altered neural circuit plasticity, dysregulation of the hypothalamic–pituitary–adrenal (HPA) axis stress response, and neuroimmune abnormalities mediated by proinflammatory cytokines. Abnormalities in GABAergic interneurons and ventral forebrain organoids derived from depressed patients have been reported, characterized by reduced dendritic complexity, altered action potential firing patterns, and decreased calcium activity (Lu et al., 2023). Modulating *HTR2C* gene expression or applying 5-HT2C receptor agonists effectively reverses abnormal neuronal activity, providing a novel model for studying depression pathogenesis and drug screening. Gong et al. (2024) utilized brain organoids to reveal that specific knockout of *WFS1* exacerbates high-fat diet-induced obesity and depression-like behaviors, while the drug riluzole—targeting this signaling pathway—reverses these abnormalities. This indicates a close connection between energy metabolic homeostasis and synaptic plasticity, suggesting that metabolic stress may directly impair neural circuit function through specific molecular pathways. Another study discovered that the circular RNA circFKBP8(5S,6) possesses protein-coding functionality; its expressed product exerts antidepressant effects by regulating the nuclear-cytoplasmic transport of glucocorticoid receptors (Jiao et al., 2024). This finding not only reveals a novel mechanism of hypothalamic–pituitary–adrenal axis dysfunction in depression but, more importantly, suggests that the circular RNA regulatory network may serve as a novel therapeutic target for depression.

## 5 Summary and prospect

Recent progress in brain organoid technology enables scientists to more accurately decipher the developmental patterns of the human brain and the pathological mechanisms of neurological disorders *in vitro*. Compared to traditional animal models, their highly humanized characteristics provide a more precise simulation of the central nervous system’s response mechanisms to external injury factors and pathological stimuli. In drug discovery, these organoids support large-scale automated screening. By constructing customized models with specific genetic backgrounds or disease phenotypes, they enable systematic evaluation of candidate drugs’ efficacy and toxicological properties. Furthermore, brain organoids demonstrate unique value in neural repair. Transplantation experiments confirm their ability to achieve functional integration with recipient brain tissue, partially reconstructing damaged neural circuits. The convergence of artificial intelligence and brain organoids holds promise for unraveling the brain’s operational mechanisms and guiding AI toward a brain-inspired computing paradigm.

However, existing *in vitro* culture systems still face several critical limitations. Firstly, brain organoids are inherently limited in scale and complexity. They cannot simulate the integrated environment of the entire body and thus are not a substitute for the human brain. Secondly, most existing organoid models capture early developmental stages but lack the maturity of adult human brain tissue. The limited synaptic complexity, immature electrophysiological properties, and incomplete epigenetic programming restrict their utility for modeling late-onset disorders. Future strategies should explore extended culture durations combined with maturation-promoting factors, electrical stimulation, and transplantation approaches to achieve more adult-like state. Thirdly, current organoid protocols exhibit significant batch-to-batch variability due to differences in stem cell lines, matrix composition, and culture conditions. This variability poses challenges for reproducibility and quantitative comparisons across studies. Implementing quality control benchmarks through single-cell RNA sequencing and electrophysiological profiling will be essential for validating model consistency. Fourthly, the absence of functional vasculature limits nutrient delivery, leads to necrotic cores, and prevents the study of neurovascular interactions. Future directions should focus on creating vascularized organoids through fusion with vascular organoids or co-culture with endothelial cells in microfluidic platforms. These advances will enable the modeling of blood–brain barrier functions and improve organoid survival and maturation. Fifthly, the deficiency of functional glial populations - particularly astrocytes, oligodendrocytes, and microglia - represents a major limitation in current models. Future research should develop better protocols including optimizing co-culture systems with pre-differentiated glial cells and improving intrinsic glial differentiation through refined patterning strategies. Simultaneously, significant challenges persist regarding ethical concerns and application safety. Currently, brain organoid research remains in its exploratory phase. With ongoing advancements in the field, brain organoids are poised to play an increasingly key role in deciphering the codes of neural function and developing effective treatments for neurological disorders.
